# The fluorescent protein iLOV as a reporter for screening of high‐yield production of antimicrobial peptides in *Pichia pastoris*

**DOI:** 10.1111/1751-7915.14034

**Published:** 2022-03-21

**Authors:** Annemette Kjeldsen, Jack E. Kay, Scott Baxter, Stephen McColm, Cristina Serrano‐Amatriain, Scott Parker, Ellis Robb, S. Alison Arnold, Craig Gilmour, Anna Raper, Graeme Robertson, Robert Fleming, Brian O. Smith, Ian G. Fotheringham, John M. Christie, Leonardo Magneschi

**Affiliations:** ^1^ Ingenza Ltd Roslin Innovation Centre Charnock Bradley Building Roslin EH25 9RG UK; ^2^ 3526 Institute of Molecular, Cell and Systems Biology College of Medical, Veterinary and Life Sciences University of Glasgow Bower Building Glasgow G12 8QQ UK; ^3^ The Roslin Institute & Royal (Dick) School of Veterinary Studies University of Edinburgh Easter Bush Midlothian EH25 9RG UK

## Abstract

The methylotrophic yeast *Pichia pastoris* is commonly used for the production of recombinant proteins at scale. The identification of an optimally overexpressing strain following transformation can be time and reagent consuming. Fluorescent reporters like GFP have been used to assist identification of superior producers, but their relatively big size, maturation requirements and narrow temperature range restrict their applications. Here, we introduce the use of iLOV, a flavin‐based fluorescent protein, as a fluorescent marker to identify *P. pastoris* high‐yielding strains easily and rapidly. The use of this fluorescent protein as a fusion partner is exemplified by the production of the antimicrobial peptide NI01, a difficult target to overexpress in its native form. iLOV fluorescence correlated well with protein expression level and copy number of the chromosomally integrated gene. An easy and simple medium‐throughput plate‐based screen directly following transformation is demonstrated for low complexity screening, while a high‐throughput method using fluorescence‐activated cell sorting (FACS) allowed for comprehensive library screening. Both codon optimization of the iLOV_NI01 fusion cassettes and different integration strategies into the *P*. *pastoris* genome were tested to produce and isolate a high‐yielding strain. Checking the genetic stability, process reproducibility and following the purification of the active native peptide are eased by visualization of and efficient cleavage from the iLOV reporter. We show that this system can be used for expression and screening of several different antimicrobial peptides recombinantly produced in *P. pastoris*.

## Graphical abstract

The methylotrophic yeast *Pichia pastoris* is commonly used for production of recombinant proteins at scale, but identification of an optimally over‐expressing strain following transformation can be time and reagent consuming. Here we introduce the use of iLOV, a flavin based fluorescent protein, as a removable fluorescent marker to identify *P. pastoris* jackpot strains easily and rapidly. We show that this system can be used for expression, screening, and monitoring of genetic stability and process reproducibility of several different antimicrobial peptides recombinantly produced in *Pichia*.

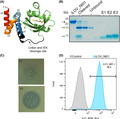

## Introduction

The methylotrophic yeast *Pichia pastoris* (syn. *Komagataella phaffi*) is a well‐established host for heterologous protein production. Its ability to grow to very high cell densities along with high‐yielding expression methods controlled by strong and tightly regulated promoters has resulted in *P. pastoris* being the second most used organism for recombinant protein expression (Ahmad *et al*., 2014; Bill, 2014), particularly for biopharmaceuticals (Vogl *et al*., 2013).

Standard methods for generating a production strain for heterologous protein production in *P*. *pastoris* involve incorporating the target gene into the genome by homologous recombination under the control of the AOX1 (alcohol oxidase 1) promoter, P_AOX1_, resulting in tightly regulated, methanol‐inducible expression (Macauley‐Patrick *et al*., 2005). Clonal variation within *P. pastoris* is well‐documented, and it is not uncommon for hundreds, if not thousands, of clones to be screened for identification of a commercially viable ‘jackpot’ strain (Aw *et al*., 2017). Much of the variation is attributed to the chromosomal integration of a variable number of copies of the expression cassette. However, there is not necessarily a linear relationship between the gene copy number and the expression yield of the target (Aw and Polizzi, 2013, 2016), making identification of jackpot clones on the basis of gene copy number unreliable and the alternatives time‐consuming. Given the recombinogenic nature of *P*. *pastoris*, problems with genetic instability may also be encountered in multicopy strains (Aw and Polizzi, 2013). Other factors such as cassette integration orientation (Schwarzhans *et al*., 2016a), co‐integration of *E. coli* host plasmid‐DNA (Schwarzhans *et al*., 2016b) and the integration strategy (targeted or random) can affect the expression yield. A recent screening study on the effect of plasmid design and integration method on recombinant protein expression found that a targeted approach yielded clonal variation spanning a six‐fold range, while the ectopically integrated cassettes spanned a 25‐fold range. A random approach to cassette integration yielded a greater phenotype variation, and an increased likelihood of identifying a high producer when a sufficient number of transformed strains was compared (Vogl *et al*., 2018).

In addition to the integration strategy, the impact of codon optimization must also be carefully considered when improving recombinant protein yield in yeast (Hu *et al*., 2013; Bill, 2014; Yang and Zhang, 2018). In *S. cerevisiae*, it has been found that the translational rates of stress‐response factors were selectively affected as cells altered their tRNA abundance depending on the growth conditions (Torrent *et al*., 2018). It is reasonable to assume that a similar mechanism would be present in *P*. *pastoris*, hence the common view that there is a single optimal codon usage frequency should be reconsidered. Comparison of the expression of non‐codon‐optimized fluorescent proteins with that of their codon‐optimized equivalents in *S. cerevisiae* showed that the protein products’ *in vivo* characteristics – specifically brightness and monomerism – altered as did the expression yield (Botman *et al*., 2019). Thus, an approach using a library of expression cassettes each with a different codon usage for the same amino acid sequence may yield a more robust and consistent expression system under a variety of conditions. The combination of a library of potential integrants and the variation seen from randomly targeted integration events that result in a range of integrated DNA copy numbers makes for a highly diverse strain library, requiring an effective screening method suitable for high numbers of clones.

The antimicrobial peptide (AMP) epidermicin NI01, originally isolated from *Staphylococcus epidermidis,* is a 51‐amino acid bacteriocin containing a high proportion of cationic and hydrophobic residues that displays potent antimicrobial activity towards a range of pathogenic Gram‐positive strains, including methicillin‐resistant *S. aureus* (MRSA) (Sandiford and Upton, 2012). It has been classified as a four‐helix bundle Type A in subclass IId of bacteriocins produced from Gram‐positive bacteria, a category that includes other promising AMPs, such as aurecin A53, lacticin Z, lacticin Q, and BacSP222 (Wladyka *et al*., 2015; Nowakowski *et al*., 2018). The mechanism of action of these AMPs is thought to be through pore‐formation in the pathogen cell membrane, causing death of the pathogen. The four‐helix bundles appear to have a C‐terminal hydrophilic face and an N‐terminal hydrophobic face, which is responsible for pathogen binding and recognition (Nowakowski *et al*., 2018). Initial studies on the antimicrobial activity of epidermicin NI01 performed using chemically synthesized peptide shows great promise as a therapeutic agent (Gibreel and Upton, 2013; Halliwell *et al*., 2017). To further develop this novel antibiotic, a more economic and scalable manufacturing method is required. Given its antimicrobial properties and membrane‐binding tendencies, NI01 is a challenging target for heterologous expression in bacterial hosts, but intracellular expression in *P*. *pastoris* may prove a fitting production method. To ensure regulatory compliance for manufacture of future biotherapeutics such as NI01, it is essential that any biopharmaceutical production route, is scalable, reproducible, and can be operated under the principles of Good Manufacturing Practice (GMP). The optimized production strain generated here has been used in a separate manufacturing process that meets these requirements.

Semi‐high‐throughput screening processes in 96‐well‐deep well plates have been developed to allow for microscale cultures of *P. pastoris* with minimal cell death and low well‐to‐well deviation (Weis *et al*., 2004). Nonetheless, a truly rapid and high‐throughput (HTP) screening method to assess recombinant protein expression level in *P. pastoris* is still missing. Fluorescent proteins are used in methods to evaluate expression levels and subcellular localization in *E. coli*, insect and mammalian cells (Su, 2005; McKenzie and Abbott, 2018). However, the use of fluorescent reporters, specifically GFP, within *P. pastoris* has been found to pose some challenges. Lenassi Zupan *et al*. (2004) noted that upon being expressed at high levels (40% of total cellular protein) GFP would spontaneously form fluorescent particles within the cytoplasm. It was concluded that GFP is able to enter into the peroxisomes and is inserted in densely packed layers of alcohol oxidase (Lenassi Zupan *et al*., 2004). The study found that these particles formed even when GFP was expressed at 10 times lower concentration (4% of total cellular protein). Great care must be taken in interpreting results when using GFP as a reporter for trafficking or targeting purposes since incorrect and misleading quantification of GFP could be obtained due to the formation of fluorescent particles containing internal material unavailable for fluorescence measurements. The formation of such particles may also hinder secretion of GFP‐fusion proteins.

A recent report on *in vivo* characterization of 27 fluorescent proteins (FP) in yeast using *S. cerevisiae* as a production host highlighted that most GFP‐derivatives are optimized for effective fluorescence at 37°C, whereas yeast is often cultured at 30°C (Botman *et al*., 2019). This study did not include any flavin‐based fluorescent proteins (FbFP) in the analysis, despite these being promising alternatives. FbFPs are derived from Light, Oxygen, or Voltage‐sensing (LOV) flavoprotein domains first identified in plant phototropins (Salomon *et al*., 2000). The improved FbFP iLOV derived from *A. thaliana* phot2 LOV2 (Chapman *et al*., 2008) has been demonstrated as an excellent fluorescent protein candidate to study protein expression in *E. coli* (Gawthorne *et al*., 2012). The flavin mononucleotide (FMN) chromophore of iLOV provides not only a bright yellow colour to the protein, allowing for easy tracking during purification, but also allows overexpressing cells to be easily detected by emitting green fluorescence upon blue light excitation. In contrast to GFP‐based FPs, FbFPs are both oxygen‐independent and exhibit stable fluorescence across wide temperature (up to 60°C) and pH (4–11) ranges (Mukherjee *et al*., 2013). Some FbFPs have already been shown to function well in both *C. albicans* and *S. cerevisiae* (Tielker *et al*., 2009; Eichhof and Ernst, 2016). The smaller size of FbFPs including iLOV (~13.5 kDa) also reduces the genetic payload compared with GFP (~30 kDa) (Chapman *et al*., 2008).

Here, we report the use of iLOV as a fluorescent aid for high‐throughput screening of a high diversity library expressed in *P. pastoris*, and thereby investigate the effect of varied codon usage and integration strategies on the protein yield of the AMP NI01. iLOV allowed for the facile selection of jackpot clones while incorporation of an enterokinase cleavage site between iLOV and NI01 enabled recovery of the native AMP sequence which retained biological activity. Genetic stability of the strains and process performance could also be monitored in real time through iLOV fluorescence. In addition to NI01, we show that this system can be used to select high‐yielding recombinant *P. pastoris* strains for a wide range of AMPs.

## Results and discussion

### iLOV as a qualitative reporter of protein production in *P. pastoris*

**Fig. 1:**
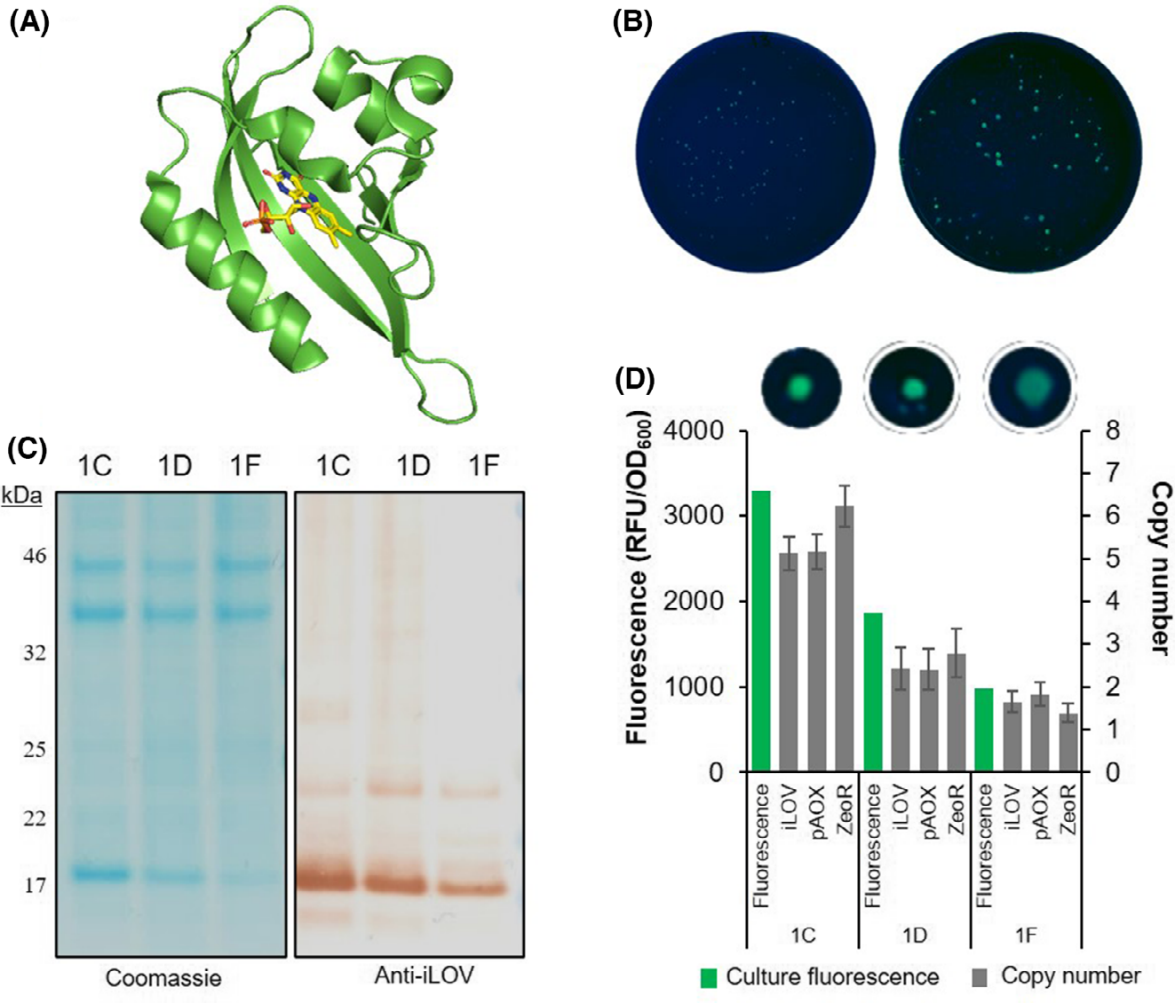
Verification of colony fluorescence corresponding to liquid culture fluorescence, copy number and iLOV expression. A. Cartoon representation of the crystal structure of iLOV (PDB: 4EES). FMN shown in stick representation (C: yellow, N: blue, O: red, P: orange). B. Replica plating of transformation plates onto induction plates containing 1% v/v methanol visualized under 302 nm light after 4 days of induction. Images of colonies from the non‐expressing negative control (left) and iLOV under the P_AOX1_ control (right) were analysed identically for variations in fluorescence intensities of the individual colonies. C. SDS‐PAGE Coomassie stained gel (left) and Western blot (anti‐iLOV antibody) (right) of the whole cell lysate from the cultures analysed in (B). D. Comparison of the colony fluorescence of three colonies, 1C, 1D and 1F (top) with normalized fluorescence in liquid culture (left‐hand axis) after 24 h induction with 0.5% v/v methanol and the integrated expression cassette copy number (right hand axis) as determined by qPCR.

To demonstrate the relationship between iLOV fluorescence, integrated copy number and iLOV protein yield in *P. pastoris*, a SacI‐linearized expression cassette containing the iLOV reporter gene under the control of P_AOX1_ promoter was integrated at the AOX1 locus in the *P. pastoris* CBS7435 MutS genome. Variation in colony fluorescence was visualized upon methanol induction on agar plates following replica plating (Fig. 1A). Three transformants, selected based on their relative colony fluorescence levels (high, medium, and low), were further evaluated. Culture fluorescence, protein expression levels and the number of integrated iLOV expression cassettes for these three transformants (Fig. 1D) were compared and demonstrated a near linear relationship between iLOV fluorescence and protein expression as assessed by SDS‐PAGE (Fig. 1C and S1, *R* ^2^ = 0.998). In the same strains, the relationship between fluorescence and the integrated copy number appeared to be less linear (*R* ^2^ = 0.975), although this observation is based on three data points only. A non‐linear relationship has been observed for other FPs (Aw and Polizzi, 2013, 2016), suggesting that each increase in cassette copy number does not necessarily yield a corresponding increase in protein expression. There is some disagreement in the literature as to the nature of the relationship between the number of integration events and the protein expression yield as some suggest a linear correlation while others do not (Vassileva *et al*., 2001; Hohenblum *et al*., 2004; Aw and Polizzi, 2013, 2016). It is very likely that the target itself plays the biggest role in this relationship and that selection of *P*. *pastoris* transformants using higher antibiotic concentration to select for the number of integrated transforming cassettes might not always result in the generation of the best production strains. The three clones screened in Fig. 1 were selected from the same transformation plate containing a relatively low concentration of Zeocin (250 µg ml^−1^), and the highest number of integrated cassettes was found to be between 5 and 6. Since *P*. *pastoris* has been reported to integrate up to 30–40 copies of an expression cassette (Aw and Polizzi, 2016), selecting iLOV‐expressing strains using a broader range of Zeocin concentrations could have led to the isolation of clones with a higher number of integrated cassettes, but not necessarily an increased variation in iLOV expression levels. Nonetheless, this plate‐based approach provided a fast and efficient medium‐throughput (MTP) screening method to identify high‐expressing clones based on iLOV fluorescence.

### Identification of a high‐yielding strain of NI01 and purification of biologically active material

**Fig. 2:**
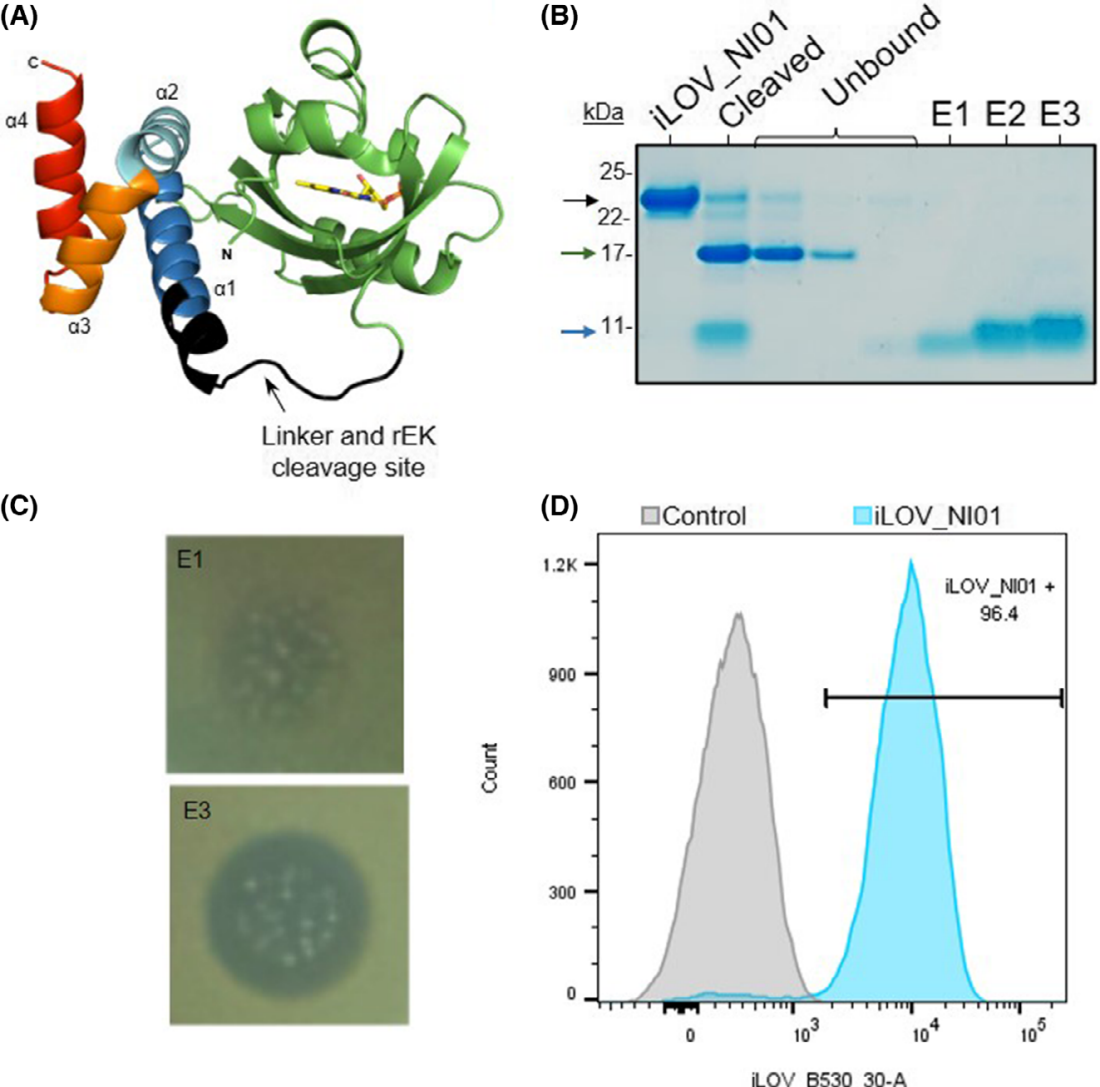
iLOV_NI01 expression analysis. A. Structure prediction of iLOV_NI01 fusion separated by the linker region and the rEK cleavage site (His‐tag at N‐term not shown). Model was generated by ColabFold (Mirdita *et al*., 2021) using the iLOV‐EK‐NI01 sequence provided in Table S5. The prediction was performed without any templates and compared with the structure of iLOV (PDB: 4EES; RMSD value = 0.38) and NI01 (PDB: 6SIG; RMSD value = 0.89). The α‐helices of NI01 and the N‐ and C‐terminal are indicated. FMN shown in stick representation (C: yellow, N: blue, O: red, P: orange). B. SDS‐PAGE analysis of purification of NI01 by CIEX. Lane 1: iLOV_NI01 following initial IMAC purification; Lane 2: Cleaved material of iLOV and NI01 following incubation with rEK; Lane 3–5: Unbound material (iLOV) flow through from CIEX purifications; Lane 6–8: Elutions 1, 2 and 3 from CIEX purification containing NI01. C. Elution fractions E1 and E3 of NI01 from the CIEX purifications showing killing activity towards *M. lutes* on impregnated agar plates. D. Expression of iLOV by *P*. *pastoris* cells with non‐expressing negative control (grey) or expressing iLOV_NI01 (blue) as determined by flow cytometry. The interval gate shows the positive population gated on the negative control.

Given the observed correlation between iLOV fluorescence and its abundance in the cultured cells, we next investigated the use of iLOV as a fluorescent reporter for the identification of high‐yielding AMP‐producing strains to address the challenges discussed above. We first performed targeted integration at the AOX1 locus with a cassette encoding the native peptide NI01 N‐terminally tagged with the fluorescent reporter iLOV. An enterokinase (rEK) cleavage site was included between the iLOV and the NI01 sequences, thus enabling cleavage of the fusion protein by rEK to yield the native, active AMP (Fig. 2A). The MTP plate‐based screen method outlined above was used to identify jackpot clones expressing high levels of iLOV_NI01. A positive correlation between culture fluorescence and protein production was observed in all strains expressing iLOV_NI01 (Fig. S2), similar to what had been established for the iLOV cassette alone (Fig. 1). A wider range of integrated copy numbers was seen in this case including a jackpot clone carrying 16 copies of the chimeric expression cassette (Strain 4H, given ID ABP269). Following induction of protein expression, cell lysis, rEK cleavage of the iLOV_NI01 fusion and separation of the fluorescent reporter from the AMP, the native NI01 peptide was obtained (Fig. 2B). This recombinant NI01 peptide showed antimicrobial activity against *M. luteus* (Fig. 2C) at levels comparable with those obtained with the chemically synthesized version of the peptide. The minimum inhibitory concentration (MIC) value determined for the iLOV_NI01 chimera was significantly higher than for the native NI01 peptide generated following rEK cleavage (Table 1), suggesting that the N‐terminal iLOV fusion can mask the biological activity of the AMP. Structural studies of NI01 have suggested that the direct α1α2 helix interaction with the target bacterial membrane is necessary for the multimodal poration mechanism of toxicity (Hammond *et al*., 2020). The iLOV fusion would hinder this interaction. While *M. luteus* growth was observed up to the tested 1.387 μM concentration of iLOV_NI01 (Table 1), growth inhibition of this test organism was observed at concentrations as low as 0.331 μM when purified NI01 was used, which is similar to the MIC of the chemically synthesized peptide.

**Table 1:** Minimum inhibitory concentration determined for iLOV_NI01 fusion and native NI01.

iLOV_NI01 (Pichia)	1.387	0.693	0.347	0.173	0.087	0.043	0.022	0.011
NI01 (Pichia)	5.295	2.648	1.324	0.662	0.331	0.165	0.083	0.041
NI01 (synthesized)	5.295	2.648	1.324	0.662	0.331	0.165	0.083	0.041

Peptide concentrations (μM) resulting in *M. luteus* growth (grey) and growth inhibition (white).

Having validated the use of iLOV fluorescence as a method for tracking the expression of an iLOV_AMP fusion protein that can be purified to yield an active AMP, we focused on the construction and identification of a strain suitable for industrial production of the recombinant NI01 peptide in a process compliant with regulatory requirements for pharmaceutical manufacturing.

### Improved NI01 production through mixed codon usage and random genomic integration, library construction and FACS

To increase the diversity of the recombinant *P. pastoris* strain library and to potentially improve the genetic stability of the final production strain, different codon usage cassettes and genomic integration strategies were tested, requiring the screening of a very large pool of transformants. Fluorescence‐activated cell sorting (FACS) provides a high‐throughput method for sorting a heterogeneous mixture of cells based upon their specific light scattering and fluorescent characteristics. To ensure that the iLOV_NI01 fusion was suitable for FACS, cell banks of the newly identified benchmark strain ABP269 described above were first investigated by flow cytometry (Fig. 2D and Fig. S3). Some cells within the ABP269 population appeared to exhibit significantly lower (~20‐fold) fluorescence than the average. While some variation in fluorescence at the population level is to be expected as a consequence of cell cycle stage and response to the inducer, genetic instability linked to the integration of contiguous repetitive expression cassettes in *Pichia pastoris* has been reported (Näätsaari *et al*., 2012; Schwarzhans *et al*., 2016b). Despite this observed variation, iLOV_NI01‐expressing cells could still be clearly distinguished from the non‐expressing control strain.

Eight new expression cassettes, in which the NI01‐specific sequence was differently codon‐optimized while the rest of the iLOV chimeric construct was left unchanged, were generated. To further increase recombinant strain diversity, two different integration strategies were employed: linearizing the plasmid within the P_AOX1_ promotor with SacI (targeted integration), or outside the expression cassette with SwaI (random integration). To test the effect of integrating multiple different expression cassettes for both integration strategies, an equimolar mixture of all 9 cassettes was used for transformation. In parallel, the individual codon‐optimized cassettes were each separately introduced via targeted integration at the AOX1 site to determine whether they drove different protein expression levels. For these experiments, the more genetically stable *P. pastoris* BSYBG11 MutS strain was used (Näätsaari *et al*., 2012; Chen *et al*., 2013; Sturmberger *et al*., 2016). The concentration of the selective agent zeocin has also been shown to affect the integrated copy number (Aw and Polizzi, 2016). Thus, independent glycerol stocks for clones selected at different Zeocin concentrations (100, 250 or 500 µg ml^−1^) were generated for mixed codon usage transformations (both targeted and random integration). In parallel, recombinants obtained from targeted integration with single codon usage cassettes were first selected on 100, 250 or 500 µg ml^−1^ zeocin plates but subsequently pooled together to generate a highly variable population to be screened by FACS. A summary of the libraries generated is shown in Table 2.

**Table 2:** Summary of FACS library.

Library ID	Plasmid stock	Digest method	Zeocin concentration
A: Targeted integration, single codon usage	Individual plasmids	SacI	Zeo_100_ + Zeo_250_ + Zeo_500_
B: Targeted integration, mixed codon usage	Mixture of 9 plasmids at equimolar ratio	SacI	Zeo_100_
			Zeo_250_
			Zeo_500_
C: Random integration, mixed codon usage	Mixture of 9 plasmids at equimolar ratio	SwaI	Zeo_100_
			Zeo_250_
			Zeo_500_

With the aim of identifying an improved *P. pastoris* biomanufacturing strain from the generated libraries, a FACS selection window was defined based on the benchmark strain ABP269, so that only strains with higher expression of the fluorescent fusion would be isolated and further characterized (Fig. S4). Cells falling into this window were sorted onto 96‐well plates containing YPD agar medium and their fluorescence levels indexed for future reference.

### Isolation of a new *iLOV_NI01* production strain

For the validation of improved *iLOV_NI01* expression in liquid culture, a total of 90 cell lines clonally expanded from single cells selected by FACS were chosen based on their fluorescence levels: from Library A (see Table 2) the five most fluorescent cell lines for each codon optimization (i.e., 45 clones) were selected; from Library B, the five most fluorescent cell lines from each Zeocin concentration (i.e., 15 clones) were chosen; 25 cell lines from Library C (to take advantage of the expected higher variability associated with random integration) and five cell lines sorted from the benchmark strain ABP269 population were also selected.

**Fig. 3:**
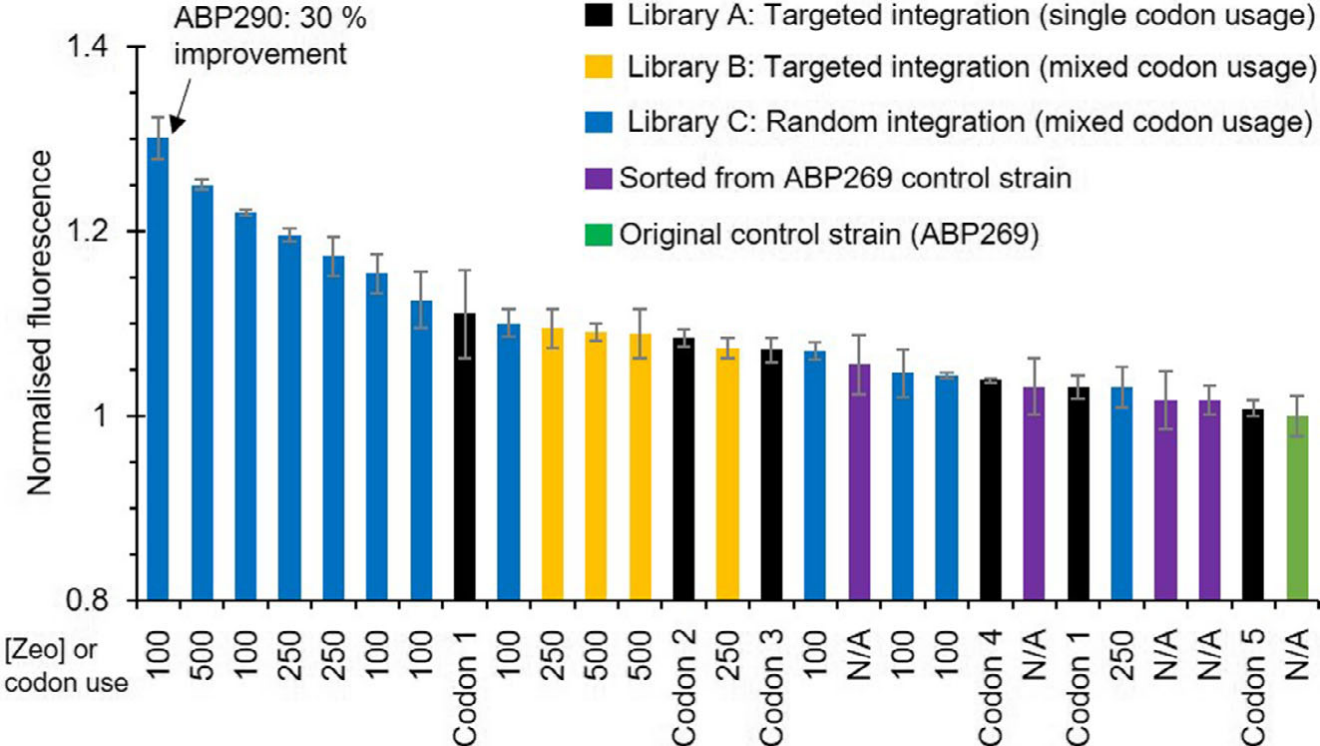
Liquid culture screen of FACS hits for improved iLOV_NI01 expression. *In vivo* fluorescence (RFU/OD_600_) of top FACS hits normalized to the original ABP269 control strain (green). Zeocin concentration (µg ml^−1^) for mixed libraries, or codon usage for single libraries, are indicated on the *x*‐axis. Control strain ABP269 had originally been selected on 250 µg ml^−1^ Zeocin.

From these 90 selected cell lines (Fig. S5 and Table S6), only 26 showed higher fluorescence in bulk liquid culture than the ABP269 reference, as shown in Fig. 3. Four of these were derived from ABP269. Several hits were identified from the single codon usage Library A (Fig. 3), although the number of integrated cassette copies was not quantified. As expected from a recombinant *P. pastoris* library where the expression cassettes have been randomly integrated into the genome (Vogl *et al*., 2018), the majority of the improved strains were found in Library C (46%). Interestingly, the top 7 strains originated from the pool with mixed codon usage and random integration. Strains from Library A accounted for 23% of the improved strains, while Library B and strains sorted from the ABP269 population each accounted for 15% of the strains showing higher fluorescence than ABP269. Based on fluorescence normalized to OD_600_ measurements, the randomly integrated strains showed improvements of between 12.5 and 30% above that of the benchmark strain ABP269, while improvements between 7 and 9% were found for targeted integration strains. These results suggest that both mixed codon usage and random genomic integration of the expression cassette are factors positively affecting overall intracellular expression of iLOV_NI01. The zeocin concentration used for selection of the original transformants did not appear to play a major role as the highest yielding strain (30% improvement) originated from the 100 µg ml^−1^ zeocin plate, while the second highest (25% improvement) was obtained from selection at 500 µg ml^−1^ zeocin. Taken together, these findings suggest that expression of recombinant iLOV protein fusions, along with high‐throughput FACS screening, can overcome limitations and bias typically associated with the selection of transformants at different zeocin concentration and potentially remove any requirement for co‐integration of an antibiotic resistance marker.

To further investigate the relationship between fluorescence and target protein yield, productivity of the most improved strain, named ABP290, was assessed at 100 ml scale relative to ABP269. Based on a comparison of the A280 absorbance of the elution peaks, ABP290 showed a 32% improvement in yield of purified NI01, in line with the observed 30% improvement in fluorescence compared with ABP269 (Table S1).

### Utility of the iLOV_NI01 fusion cassette for real‐time monitoring of strain genetic stability and process reproducibility

Genetic instability of multicopy strains due to intergenic recombination between identical contiguous heterologous DNA sequences in *P. pastoris* poses a major problem (Zhu *et al*., 2009). Recombinants isolated based on the highest initial productivity can also include those most likely to show genetic instability, whereby recombination events between contiguous identical copies of the integrated target gene leads to removal (deletion) of gene copies with corresponding losses in productivity of the transformant to produce the target protein. Genetic instability is undesirable in the establishment of a stable recombinant strain, particularly for pharmaceutical production to current Good Manufacturing Practice (cGMP) quality standards where strain stability is a priority. Since expression of iLOV protein fusions can be readily tracked by means of fluorescence levels, we investigated whether this system could also be used to monitor strain stability and productivity throughout bioprocess development and manufacturing of the target protein.

**Fig. 4:**
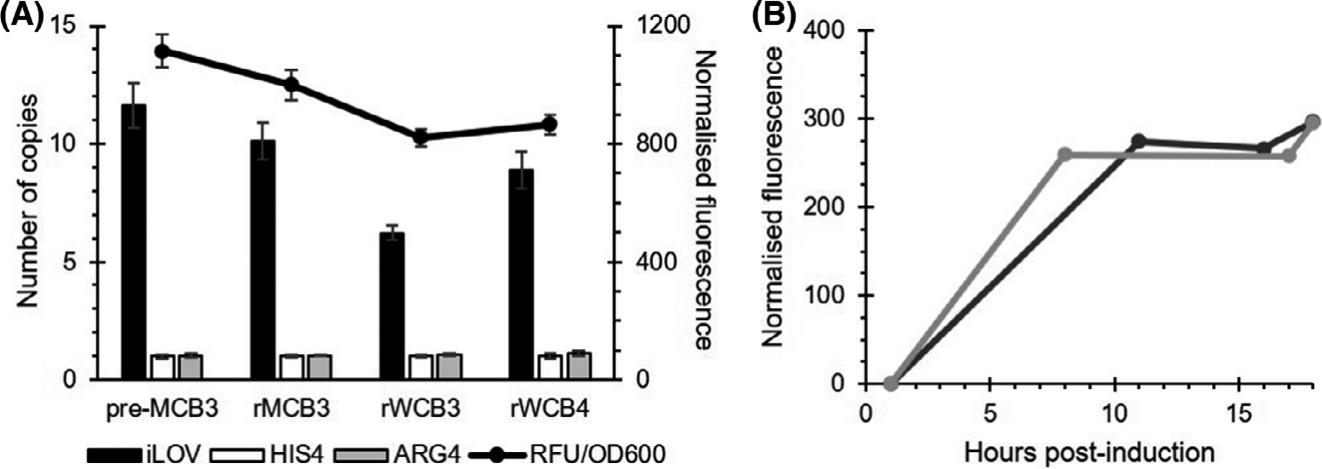
Stability of iLOV_NI01 in ABP290 and reproducibility of fermentations. A. iLOV integrants copy number quantified via qPCR (left‐hand axis) relative to the two reference controls HIS4 and ARG4, which are each present in a single copy in the *P. pastoris* genome. Culture fluorescence values at 24 h after methanol induction were normalized to OD_600_ (right hand axis). B. Reproducibility and iLOV_NI01 production in two independent fermentations monitored by fluorescence resulted in comparable recovery of the chimeric protein.

Commercial development of cell banks typically involves production of a master cell bank (MCB) obtained from a starter culture (pre‐MCB). From this MCB, working cell banks (WCB) are produced to ensure consistency of the manufacturing process. For research (r) purposes, ABP290 pre‐master cell banks (pre‐MCB3), research master (rMCB3) and research working cell banks (rWCB3 and rWCB4) were generated and their genetic stability and productivity monitored through intracellular fluorescence associated with iLOV_NI01 expression (Fig. 4A). As expected, a degree of genetic instability was observed for this strain, with a decrease from 12 (pre‐MCB3) to 9 (rWCB4) copies of the integrated iLOV_NI01 cassette. The variation in copy number between cultures established from different cell banks correlated with their observed levels of iLOV fluorescence when induced with methanol. Although both derived from rMCB3, rWCB3 (6 iLOV_NI01 integrated cassettes) and rWCB4 (10 iLOV_NI01 integrated cassettes) showed significantly different fluorescence levels, in line with the number of integrated cassettes (Fig. 4A), supporting the use of iLOV to track strain genetic stability. In addition, iLOV fluorescence allowed the facile monitoring and assessment of reproducibility of two fermentation runs that were used for the generation of the material needed to advance NI01 pre‐clinical studies (Fig. 4B). Relative final iLOV_NI01 titres closely matched the observed fluorescence levels normalized to the cultures’ OD_600_. Being able to follow the protein target production in real time is beneficial for process development enabling rapid decision making at each step of the process.

### iLOV as a universal reporter for recombinant AMP production in *P. pastoris*

To demonstrate the applicability of iLOV as a fluorescent reporter for recombinant AMP production in *P. pastoris*, we applied our screening method to seven additional AMPs that exhibit a variety of degrees of amino acid sequence identity, charge, activity profile, and post‐translational modifications, when compared with NI01 (Table S2). These AMPs were selected from the literature and include LacQ (Yoneyama *et al*., 2009), TE8 (Kumar *et al*., 2017), A53 (Netz *et al*., 2002), LL‐37 (Duplantier and van Hoek, 2013; Engelberg and Landau, 2020), His5 (Mochon and Liu, 2008), Thn1 (Ma *et al*., 2019) and DCD‐1L (Schittek *et al*., 2001; Nguyen *et al*., 2017).

For each of the iLOV_AMP constructs, *P*. *pastoris* transformations using targeted integration with single codon usage cassettes and subsequent FACS analysis were performed as described for Library A (Table 2). While a lower degree of variability is expected when using targeted rather than random integration, multiple tandem‐integrations of the cassette or the complete vector still account for significant clone‐to‐clone variability (Schwarzhans *et al*., 2016a).

**Fig. 5:**
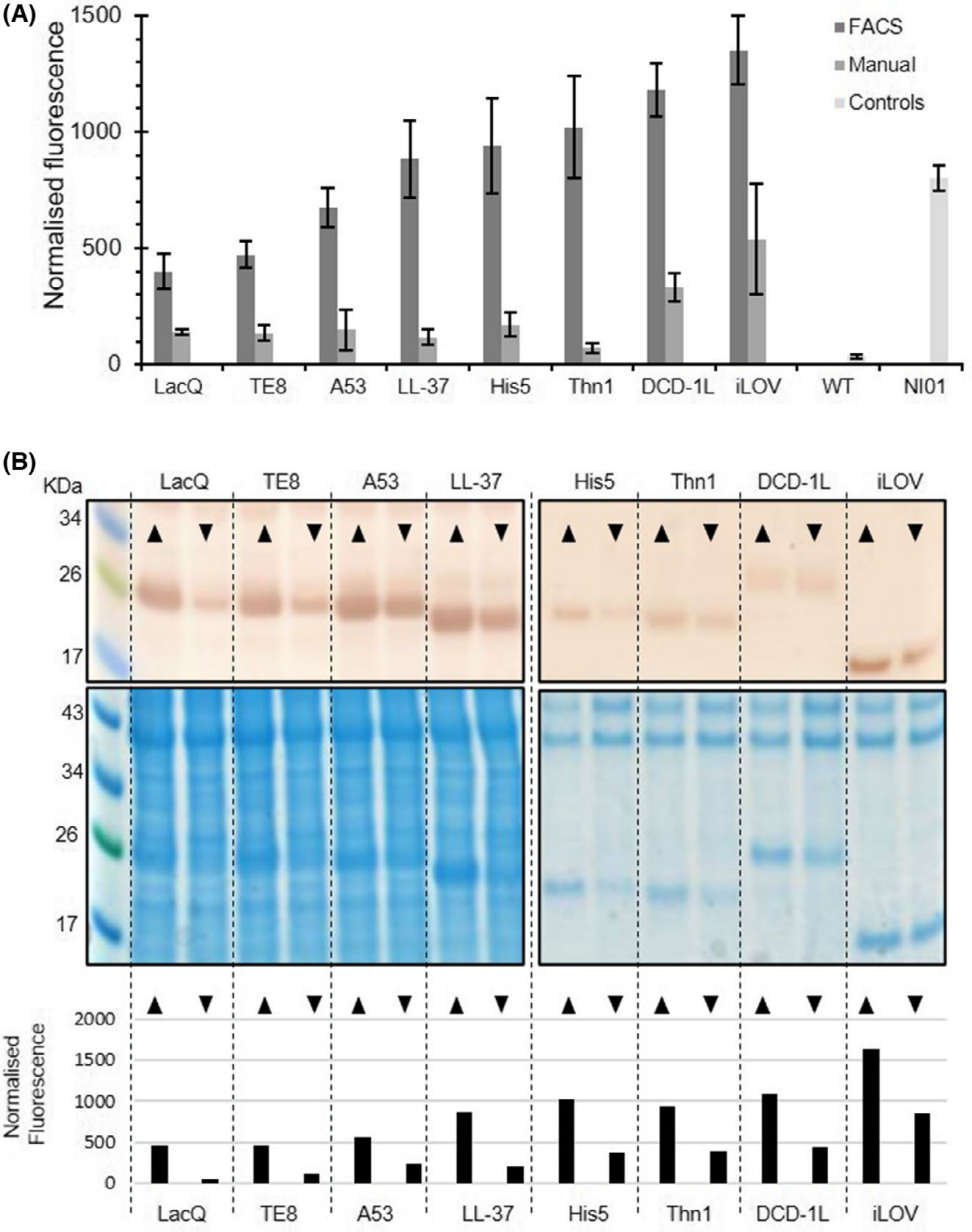
Expression comparison of iLOV_AMP constructs. A. Culture fluorescence of strains isolated by FACS compared with those manually picked at random. Average normalized fluorescence from 10 clones and associated standard deviation are shown for each set. B. Western blot (anti‐iLOV) analysis of expression levels (top) compared with normalized culture fluorescence of highest (▲) and lowest (▼) fluorescent clones selected by FACS (bottom). SDS‐PAGE Coomassie stained gels are provided as a loading control for each batch of western blotting (middle).

In addition to the FACS screen, 10 transformants for each iLOV_AMP cell line were randomly picked from plates containing 100 μg ml^−1^ Zeocin. iLOV_AMP expression levels in randomly selected and FACS isolates were compared by *in vivo* fluorescence and highest and lowest expressing strains were identified (Fig. 5). For each of the iLOV_AMP constructs tested, a higher level of fluorescence was observed for strains obtained from FACS selection, confirming previous results obtained on iLOV_NI01 showing that the combination of iLOV and FACS allows rapid identification of jackpot clones. Western blot analysis for expression levels of the iLOV_AMP fusions showed protein amounts to closely match the fluorescence levels (Fig. 5B) confirming that *in vivo* fluorescence is an effective measure of iLOV_AMP protein expression levels. As for iLOV_NI01, the combination of iLOV and FACS allows rapid identification of jackpot clones for all seven AMPs tested.

Fusion protein from the highest expressing clones for each construct were further purified from small‐scale cultures using Ni:NTA resin (Fig. S6). A high purity product (estimated > 95% pure) corresponding to the correct molecular weight was recovered for five of the seven iLOV_AMP fusion constructs after a single step purification. The exceptions were DCD‐1L and His‐5 for which the protein eluted from the IMAC step included a significant proportion of species of lower than expected molecular mass suggesting that they were subject to partial proteolysis at sites in the AMP.

To demonstrate the general applicability of the enterokinase cleavage as a method to produce native AMPs, we selected the iLOV_LL37 peptide, which is evolutionarily unrelated to NI01. The enterokinase cleavage step led to the release of a polypeptide corresponding to the correct size of native LL37 (Fig. S7). Overall, the strategy of using iLOV as a reporter for increased AMP production proved successful for a range of targets.

## Conclusion

This work demonstrates a simple and effective procedure to identify jackpot clones for AMP production in *P*. *pastoris*. The use of the FbFP iLOV as a fluorescent reporter should impose a lower cellular burden than that of GFP or other larger FPs, while still allowing direct monitoring of protein expression levels via fluorescence. Both a medium‐throughput method, mostly applicable to low complexity screening, and a high‐throughput method using FACS, which allowed the screening of more complex libraries, were demonstrated. Strain stability at cell banking and production stages can also be easily monitored, resulting in significant savings of operator time and consumables. When tested on seven additional AMP‐targets with varying degrees of amino acid sequence similarity to NI01, the method was demonstrated to be suitable for the rapid identification of high‐yielding jackpot strains. Incorporation of an enterokinase recognition site between the iLOV reporter and the AMP allows for efficient cleavage of the FbFP to yield the native protein target. The genetic stability of production strains as well as process efficiency can be monitored in real time, enabling improvements in fermentation conditions and final product yield.

We obtained reproducible (*n* = 5) yield in excess of 100 mg l^−1^ of NI01 at > 95% purity post‐maturation. The process economic calculation indicates comparable or superior manufacturing costs versus chemically synthesized NI01, but with significantly superior chemical purity. Peptides of over 50 amino acids in length are typically subject to the presence of very closely related impurities (e.g. truncated versions) when prepared by solid‐phase chemical synthesis. The recombinant biomanufacture of such products therefore offers significant advantages for clinical production at comparable yields. This process has been used to generate in excess of 20 g of active NI01 to facilitate pre‐clinical studies.

The platform described in this work is currently being used at Ingenza for isolation of superior *P. pastoris* recombinant strains expressing a wide range of iLOV‐target fusions, either intracellularly or secreted to the culture media. Further comparative work is needed between GFP and iLOV to determine iLOV aggregation status *in vivo* and whether this impacts its fluorescent read out.

## Experimental procedures

### Plasmid and strain construction

iLOV_AMP fusion cassettes were cloned into methanol‐inducible pPpT4 plasmid backbones (University of Graz, Austria) via EcoRI/NotI sites. iLOV, NI01 and alternative AMP coding sequences were optimized for expression in *P*. *pastoris* by Twist Biosciences. Different NI01 codon‐optimizations (Genewiz, IDT, Twist, GeneArt, Eurofins, BioWorks) were tested. Sequence‐verified plasmids were linearized by SacI for targeted P_AOX1_ integration, or SwaI for random integration. Following purification by gel electrophoresis, expression cassettes were introduced via electroporation into *P*. *pastoris* CBS7435 or *P*. *pastoris* BSBG11 cells. Empty vector control cell lines were also generated and used as a reference for cell autofluorescence as shown in Fig. S5. Following electroporation, cells were allowed to recover in YPS + 2% dextrose for 3 h at 30°C and then split three ways onto YPDS agar plates with 100, 250 or 500 µg ml^−1^ Zeocin and incubated at 30°C for 72 h.

### Plate‐based screening

Following transformation, plates containing distinct single colonies were selected for duplicate plating onto induction plates made up from 1.5% w/v agar, 1×YNB and 1% v/v methanol. The cells were transferred from the transformation plate onto the induction plate using sterile Whatman filter papers. Both plates were incubated at 30°C for 48 h, after which the transformation plates were stored at 4°C, while 200 µl of 50% v/v methanol was added to the lid of the induction plate and allowed to transfer to the colonies by evaporation. After a further 24 h of incubation, pictures of the induced colonies were taken illuminated by a Syngene GelVue (GVM20, 302 nm) blue light using a light box and a Samsung Galaxy S7 phone. Brightness and colour saturation of all images including a non‐expressing control were adjusted using Fiji ImageJ to allow easy identification of the most fluorescent colonies.

### Liquid culture screen

Colonies were picked from the original transformation plate that had not been exposed to blue light/UV‐illumination and resuspended in 1.75 ml BMGY in a 24‐well plate (square wells) and allowed to grow at 30°C, 250 rpm over 48 h. The plates were centrifuged at 3000 rpm for 10 min and supernatant discarded. Cells were resuspended in 1.75 induction media BMMY with 0.5% v/v methanol and incubated at 30°C, 250 rpm for 48 h. The OD_600_ and fluorescence at 450 nm excitation, 500 nm emission was measured in a black‐walled, clear F‐bottom 96‐well plate (Costar) using a FLUOstar Omega Microplate Reader (BMG LABTECH, Ortenberg, Germany).

### SDS‐PAGE and Western blotting

Whole cell lysate samples were prepared by normalizing the cell density by OD_600_ and resuspending cell pellets in Bolt™ 1×LDS Sample buffer with 0.9% w/v dithiothreitol (DTT) and boiled for 5 min. Proteins were resolved on a SDS 4–12% BisTris polyacrylamide gel (Novex) using the Bolt™ system following the standard procedure recommended by the manufacturer. Color Prestained Protein Standard, Broad Range (NEB) was used as molecular weight marker. For Coomassie staining, gels were stained using InstantBlue™ (Expedeon, Heidelberg, Germany). The percentage of iLOV and iLOV_NI01 expressed was analysed using Fiji ImageJ relative to the total amount of protein detected by staining. Briefly, the full lane of each sample was plotted as a histogram, and the area under the curve for each peak estimated. The peak corresponding to the gene of interest was divided by the sum of all peaks within the lane for a relative comparison. For western blot, proteins were transferred onto PVDF membranes after SDS‐PAGE using the iBlot 2 Dry Blotting System (Thermo Fisher, Waltham, MA, USA). The membrane was blocked using 5% milk in PBS‐Tween for 1 h before incubation with primary antiserum (rabbit‐anti‐phiLOV2.1, John Christie lab) at a 1:2500 dilution. After washing, the membrane was incubated with secondary antiserum (goat‐anti‐rabbit HRP conjugate, Fisher Scientific) at 1:25000 dilution. The antibody was visualized by DAB staining in stable peroxide buffer (Thermo Fisher).

### Copy number determination by qPCR

Genomic DNA extraction was performed on 1 ml BMGY culture grown for 48 h using the YeaStar Genomic DNA kit (Zymo Research, Irvine, CA, USA) following the manufacturer’s instructions. The DNA was quantified using the Qubit 2.0^®^ system, and quality checked by capillary electrophoresis using a TapeStation System (Agilent Technologies, Santa Clara, CA, USA). Primers used are reported in Table S3. qPCR was performed using PowerUp™ SYBR™ Green Master Mix (Applied Biosystems™) following the conditions recommended by the manufacturer. Reactions were run in triplicate on a PikoReal™ Real‐Time PCR System (Thermo Scientific™). Data was analysed by the 2^−ΔΔCq^ method.

### Purification of NI01 and AMP

About 100 ml cultures of *P*. *pastoris* carrying the gene of interest were grown in BMGY medium for 72 h at 30°C, 250 rpm. Cells were harvested by centrifugation at 5000 rpm for 20 min in 50 ml tubes. Subsequently, cells were resuspended in 100 ml BMMY media containing 0.5% v/v methanol to induce expression. The culture was incubated for 24 h at 30°C, 250 rpm and the cells harvested by centrifugation as above. Strain growth and protein production runs were carried out using a high cell density fed‐batch fermentation of *P*. *pastoris* using proprietary growth conditions and a minimal medium. Samples were taken at regular intervals throughout the course of the fermentation and analysed for biomass, protein expression, fluorescence, and key metabolite concentrations. For lysis, cells were resuspended in a proprietary chemical lysis buffer and lysed overnight. Clarified cell lysate was obtained by centrifugation at 12 000 rpm at 4°C for 30 min.

The lysate was incubated with Ni‐NTA resin (Invitrogen, Waltham, MA, USA) for IMAC purification of iLOV_NI01. The protein was eluted with 50 mM TrisHCl, pH 8.0, 150 mM NaCl, 250 mM imidazole. Fractions containing iLOV_NI01 were incubated with rEK (30 µg ml^−1^, Ingenza Ltd, Roslin, UK) overnight. The cleaved NI01 was applied to a SP Sepharose FF column using an ÄKTA Pure150 System (GE Healthcare, Chicago, IL, USA) and eluted using gradient of increasing proportion of elution buffer (50 mM CHES, pH 9.5, 1 M NaCl) to 100% over 20 column volumes. The purified NI01 was quantified by integrating the absorbance peak at 280 nm using the UNICORN software. Alternate AMPs were purified by harvesting 15 ml expression cultures (BMGY/BMMY) and lysis with YeastBuster™ commercial lysis reagent (Sigma Aldrich, St. Louis, MO, USA). Lysis reactions (at 150 g WCW/L of lysis buffer) were incubated at 30°C, 250 rpm overnight, before clarification (12 000 rpm at 4°C for 30 min). Six ml of CFE were applied to a 5 ml HisTrap FF crude column at a flow velocity of 30 cm h^−1^. The column was washed with five column volumes of wash buffer (50 mM Tris:HCl pH 7.5) before the target AMP was eluted with elution buffer 1 (50 mM Tris:HCl pH 7.5 + 100 mM Imidazole; Late elution: 50 mM Tris:HCl pH 7.5 + 400 mM Imidazole), followed by elution buffer 2 ((50 mM Tris:HCl pH 7.5 + 1 M Imidazole).

### Activity test of NI01

Purified NI01 was tested for antimicrobial activity by a spot assay onto agar plates impregnated with *Micrococcus luteus*. Briefly, 5 ml of LB was inoculated with *M. luteus* in a 50 ml falcon tube, grown overnight at 37°C, 250 rpm and added to warm (42°C) LB agar to a final OD_600_ 0.1. Agar plates were poured and allowed to dry before 10 µl of each elution to be tested was spotted onto the plate. Plates were incubated at 37°C overnight. Active NI01 was identified where no growth of *M. luteus* was observed in the agar.

Minimum Inhibitory Concentration (MIC) was determined by CLSI standardized broth micro‐dilution method using *Micrococcus luteus* as the test organism (Weinstein *et al*., 2018). Briefly, a suspension of *M. luteus* was prepared in 0.85% (w/v) NaCl at OD 0.08–0.12, corresponding to a microbial suspension of 1–2 × 10^8^ CFU ml^−1^. A 150‐fold dilution was made in Mueller Hinton Broth (MHB) to result in a microbial suspension of approximately 10^6^ CFU ml^−1^. In a 96‐well round bottom plate, 50 μl of this suspension was added to each well containing 50 μl of NI01 dilutions at the appropriate concentration (0.25–32 μg ml^−1^) prepared using MHB as a diluent. Plates were sealed with a breathable seal and placed in the static incubator at 37°C for 24 h. MIC was determined after visual inspection and defined as the lowest concentration of NI01 where no *M. luteus* growth is observed.

### Flow cytometry and FACS

BMGY cultures of iLOV_AMP expressing and WT *P. pastoris* strains were grown for 3 days at 30°C, 250 rpm. The cultures were centrifuged at 1122 *g* for 5 min and supernatant discarded. The cells were resuspended in BMMY (0.5% v/v methanol) and incubated at 30°C, 250 rpm for approximately 20 h. Samples for flow cytometry were prepared by dilution of cells to OD_600_ 0.1 in BMMY; for FACS analysis, samples were normalized to an OD_600_ equal to 0.75. Flow cytometry samples were acquired on a BD LSRFortessa X20, 3‐laser/14 detector cytometer, using BD FACSDiva software version 9.0 (BD). Single‐cell sorting was performed using an 85 μm nozzle on a BD FACS Aria IIIu, 4‐laser/11 detector cell sorter using BD FACSDiva software version 8.0.1 (BD). The configuration for both cytometers is listed in Table S4. Data were analysed using FlowJo version 10.5.3 (BD). The gating strategies applied are detailed in Figs S3 and S4. FACS samples were sorted onto YPD agar in 96‐well plates and incubated at 30°C for 3 days to form colonies.

## Conflict of interest

I.F. is a director of Amprologix, a biotechnology company developing the NI01/Epidermicin molecule as a potential therapeutic. Ingenza has filed Patent Application 16/952, 983US protecting the use of the iLOV as a tool to detect heterologous gene expression in *P. pastoris*.

## Supporting information


**Table S1.** Relationship between iLOV_NI01 fluorescence normalised by OD_600_ and NI01 protein yield in strain ABP290 relative to ABP269.
**Table S2.** Sequences of ABPs.
**Table S3.** Primers used for qPCR.
**Table S4.** Cytometer configuration.
**Table S5.** List of the iLOV‐linker‐Enterokinase‐Site‐AMP amino acidic sequences (AMPs sequences highlighted in grey)
**Table S6.** Strains performance summary. Production yield of NI01 was determined for production strains ABP290 and the ABP269 control (in bold).
**Fig. S1.** Relationship between iLOV fluorescence, expression and integration events in *P. pastoris*.
**Fig. S2.** MTP method for identifying a *P. pastoris* strain expressing iLOV_NI01.
**Fig. S3.** Gating strategy used for identification of iLOV postive cells.
**Fig. S4.** Representative gating strategy for single cell selection of clones with improved expression of iLOV.
**Fig. S5.** Full results from liquid screen of FACS hits.
**Fig. S6.** SDS‐PAGE analysis of IMAC purifications of His‐tagged iLOV and iLOV_AMP fusions.
**Fig. S7.** SDS‐PAGE analysis of rEK activity against iLOV and iLOV_LL37.
