# Supplementary material for: The fluorescent protein iLOV as a reporter for screening of high‐yield production of antimicrobial peptides in Pichia pastoris
Source: Microb Biotechnol. 2022 Mar 21;15(7):2126–39. doi: 10.1111/1751-7915.14034 (PMC9249318; doi:10.1111/1751-7915.14034)

Table S1. Relationship between iLOV_NI01 fluorescence normalised by OD_600_ and NI01 protein yield in strain ABP290 relative to ABP269.

| **Strain** | **RFU/OD600** | **NI01 yield** |
| --- | --- | --- |
| ABP269 | 100% | 100% |
| ABP290 | 130% | 132% |

Table S2. Sequences of ABPs.

| **ABP** | **Origin** | **Sequence** | **% Identity to NI01** |
| --- | --- | --- | --- |
| **NI01** | *S. epidermis* | MAAFMKLIQFLATKGQKYVSLAWKHKGTILKWINAGQSFEWIYKQIKKLWA | N/A |
| **TE8** | *S. capitis* | MAGFMKLIQFLATKGQKYVSLAWKHKGTILKWINAGQSFEWIYKQIKKLWS | 97% |
| **LacQ** | *L. lactis* | MAGFLKVVQLLAKYGSKAVQWAWANKGKILDWLNAGQAIDWVVSKIKQILGIK | 51% |
| **A53** | *S. aureus* | MSWLNFLKYIAKYGKKAVSAAWKYKGKVLEWLNVGPTLEWVWQKLKKIAGL | 36% |
| **LL-37** | *H. sapiens* | LLGDFFRKSKEKIGKEFKRIVQRIKDFLRNLVPRTES | 21% |
| **His5** | *H. sapiens* | DSHAKRHHGYKRKFHEKHHSHRGY | 30% |
| **DCD-1L** | *H. sapiens* | SSLLEKGLDGAKKAVGGLGKLGKDAVEDLESVGKGAVHDVKDVLDSVL | 17% |
| **Thn1** | *P. maculiventris* | GSKKPVPIIYCNRRTGKCQRM | 16% |

Table S3. Primers used for qPCR.

| iLOV Fw | GCCGTGAAGAAATTCTGGGTC |
| --- | --- |
| iLOV Rev | CGCTGATCACGAATGGCATC |
| NI01 Fw | CAATTCTTGGCTACTAAGGGTCA |
| NI01 Rev | CTCGAATGACTGTCCTGCGT |
| ZEO-R Fw | TGCTCGTGATGTTGCTGGTG |
| ZEO-R Rev | CAACACCGGCAAAGTCGTCC |
| pAOX1 Fw | TGCCATCCGACATCCACAGG |
| pAOX1 Rev | CAACGGTCTGCTGCTAGTGT |
| HIS4 Fw | TTTGACTACTGACCGCCCCG |
| HIS4 Rev | ACGAGTACACCAGGCCCAAC |
| ARG4 Fw | GCAGAGTGGGCAGAAGGGAA |
| ARG4 Rev | ACTCACCCAAGCGACGTTCA |
| OCH1 Fw | GCCGTTTCTGTCATTTGCGT |
| OCH1 Rev | AGTGATCGGAGCGTCAATGGA |

Table S4. Cytometer configuration.

|  | Laser | Channel | Voltage |
| --- | --- | --- | --- |
| BD LSRFortessa X20 | 488nm | FSC-A | 513 |
|  |  | SSC-A | 371 |
|  |  | B 530/30 | 418 |
| BD FACS Aria IIIu | 488nm | FSC-A | 513 |
|  |  | SSC-A | 371 |
|  |  | B 530/30 | 418 |

FSC-A (forward scatter area) indicates cells size; SSC-A (side scatter area) indicates cells granularity; filter B530/30 was used to collect light from cells which have been excited by the 488nm laser and emitted light between 515-545nm wavelength.

Table S5. List of the iLOV-linker-Enterokinase-Site-AMP amino acidic sequences (AMPs sequences highlighted in grey)

| **>iLOV-EK-TE8**  MGHHHHHHHHMATTLERIEKNFVITDPRLPDNPIIFASDGFLELTEYSREEILGRNARFLQGPETDQATVQKIRDAIRDQRETTVQLINYTKSGKKFWNLLHLQPVRDQKGELQYFIGVQLDGTEHVGSGSGSGDDDDKMAGFMKLIQFLATKGQKYVSLAWKHKGTILKWINAGQSFEWIYKQIKKLWS |
| --- |
| **>iLOV-EK-LacQ**  MGHHHHHHHHMATTLERIEKNFVITDPRLPDNPIIFASDGFLELTEYSREEILGRNARFLQGPETDQATVQKIRDAIRDQRETTVQLINYTKSGKKFWNLLHLQPVRDQKGELQYFIGVQLDGTEHVGSGSGSGDDDDKMAGFLKVVQLLAKYGSKAVQWAWANKGKILDWLNAGQAIDWVVSKIKQILGIK |
| **>iLOV-EK-A53**  MGHHHHHHHHMATTLERIEKNFVITDPRLPDNPIIFASDGFLELTEYSREEILGRNARFLQGPETDQATVQKIRDAIRDQRETTVQLINYTKSGKKFWNLLHLQPVRDQKGELQYFIGVQLDGTEHVGSGSGSGDDDDKMSWLNFLKYIAKYGKKAVSAAWKYKGKVLEWLNVGPTLEWVWQKLKKIAGL |
| **>iLOV-EK-LL-37**  MGHHHHHHHHMATTLERIEKNFVITDPRLPDNPIIFASDGFLELTEYSREEILGRNARFLQGPETDQATVQKIRDAIRDQRETTVQLINYTKSGKKFWNLLHLQPVRDQKGELQYFIGVQLDGTEHVGSGSGSGDDDDKLLGDFFRKSKEKIGKEFKRIVQRIKDFLRNLVPRTES |
| **>iLOV-EK-His5**  MGHHHHHHHHMATTLERIEKNFVITDPRLPDNPIIFASDGFLELTEYSREEILGRNARFLQGPETDQATVQKIRDAIRDQRETTVQLINYTKSGKKFWNLLHLQPVRDQKGELQYFIGVQLDGTEHVGSGSGSGDDDDKDSHAKRHHGYKRKFHEKHHSHRGY |
| **>iLOV-EK-DCD-1L**  MGHHHHHHHHMATTLERIEKNFVITDPRLPDNPIIFASDGFLELTEYSREEILGRNARFLQGPETDQATVQKIRDAIRDQRETTVQLINYTKSGKKFWNLLHLQPVRDQKGELQYFIGVQLDGTEHVGSGSGSGDDDDKSSLLEKGLDGAKKAVGGLGKLGKDAVEDLESVGKGAVHDVKDVLDSVL |
| **>iLOV-EK-Thn1**  MGHHHHHHHHMATTLERIEKNFVITDPRLPDNPIIFASDGFLELTEYSREEILGRNARFLQGPETDQATVQKIRDAIRDQRETTVQLINYTKSGKKFWNLLHLQPVRDQKGELQYFIGVQLDGTEHVGSGSGSGDDDDKGSKKPVPIIYCNRRTGKCQRM |
| **>iLOV-EK-NI01**  MGHHHHHHHHMATTLERIEKNFVITDPRLPDNPIIFASDGFLELTEYSREEILGRNARFLQGPETDQATVQKIRDAIRDQRETTVQLINYTKSGKKFWNLLHLQPVRDQKGELQYFIGVQLDGTEHVGSGSGSGDDDDKMAAFMKLIQFLATKGQKYVSLAWKHKGTILKWINAGQSFEWIYKQIKKLWA |

Table S6. Strains performance summary. Production yield of NI01 was determined for production strains ABP290 and the ABP269 control (in bold).

| **Library code** | **Strain ID** | **Zeo selection concentration (µg/mL)** | **Fluorescence / OD (%)** | **Production yield of NI01 (% relative to ABP269)** |
| --- | --- | --- | --- | --- |
| C | **ABP290** | 100 | 130 | 132 |
|  | ABP284-G2 | 500 | 125 |  |
|  | ABP282 PL1-C7 | 100 | 122 |  |
|  | ABP283-D2 | 250 | 120 |  |
|  | ABP283-E1 | 250 | 117 |  |
|  | ABP282 PL2-E12 | 100 | 115 |  |
|  | ABP282 PL3-H3 | 100 | 113 |  |
| A | ABP275-G2 | Pooled | 111 |  |
| C | ABP282 PL1-B7 | 100 | 110 |  |
| B | ABP280-A6 | 250 | 109 |  |
|  | ABP281-E7 | 500 | 109 |  |
|  | ABP281-C2 | 500 | 109 |  |
| A | ABP277-H6 | Pooled | 108 |  |
| B | ABP280-E7 | 250 | 107 |  |
| A | ABP273-H5 | Pooled | 107 |  |
| C | ABP282 PL2-H3 | 100 | 107 |  |
| N/A | ABP269-A1 | N/A | 106 |  |
| C | ABP282 PL2-A12 | 100 | 105 |  |
|  | ABP282 PL1-B10 | 100 | 104 |  |
| A | ABP276-F2 | Pooled | 104 |  |
| N/A | ABP269-A8 | N/A | 103 |  |
| A | ABP275-A5 | Pooled | 103 |  |
| C | ABP283-A3 | 250 | 103 |  |
| N/A | ABP269-E2 | N/A | 102 |  |
|  | ABP269-F1 | N/A | 102 |  |
| A | ABP278-F10 | Pooled | 101 |  |
| N/A | **ABP269** | N/A | 100 | 100 |
| A | ABP278-G1 | Pooled | 99 |  |
|  | ABP270-H10 | Pooled | 99 |  |
| N/A | ABP269-H3 | N/A | 98 |  |
| A | ABP274-G3 | Pooled | 97 |  |
| C | ABP282 PL3-H4 | 100 | 97 |  |
| A | ABP277-A9 | Pooled | 97 |  |
| B | ABP281-B8 | 500 | 97 |  |
|  | ABP280-F10 | 250 | 96 |  |
| A | ABP272-H4 | Pooled | 96 |  |
| C | ABP282 PL3-B7 | 100 | 96 |  |
|  | ABP282 PL1-F9 | 100 | 95 |  |
|  | ABP284-D5 | 500 | 93 |  |
|  | ABP282 PL2-A5 | 100 | 93 |  |
|  | ABP283-D10 | 250 | 92 |  |
|  | ABP283-H2 | 250 | 91 |  |
| A | ABP275-A3 | Pooled | 91 |  |
|  | ABP270-D4 | Pooled | 91 |  |
| B | ABP281-C9 | 500 | 89 |  |
| A | ABP273-D8 | Pooled | 89 |  |
| C | ABP284-B1 | 500 | 89 |  |
| B | ABP281-H5 | 500 | 88 |  |
| A | ABP278-F5 | Pooled | 88 |  |
|  | ABP274-D9 | Pooled | 88 |  |
|  | ABP270-A2 | Pooled | 87 |  |
|  | ABP278-C3 | Pooled | 86 |  |
| C | ABP282 PL2-F2 | 100 | 86 |  |
| A | ABP270-G11 | Pooled | 86 |  |
|  | ABP273-E8 | Pooled | 85 |  |
| B | ABP279-B1 | 100 | 85 |  |
| A | ABP272-E1 | Pooled | 83 |  |
| B | ABP279-C11 | 100 | 83 |  |
| A | ABP270-G12 | Pooled | 82 |  |
|  | ABP275-A2 | Pooled | 81 |  |
| C | ABP284-F12 | 500 | 81 |  |
|  | ABP282 PL3-H5 | 100 | 81 |  |
| A | ABP272-D4 | Pooled | 80 |  |
|  | ABP277-H8 | Pooled | 80 |  |
|  | ABP274-G7 | Pooled | 79 |  |
|  | ABP277-E6 | Pooled | 79 |  |
|  | ABP276-A12 | Pooled | 78 |  |
|  | ABP276-G5 | Pooled | 78 |  |
|  | ABP273-F12 | Pooled | 77 |  |
|  | ABP271-E9 | Pooled | 75 |  |
|  | ABP271-G3 | Pooled | 75 |  |
|  | ABP278-C8 | Pooled | 75 |  |
| C | ABP284-D3 | 500 | 74 |  |
| A | ABP271-D9 | Pooled | 74 |  |
|  | ABP273-H9 | Pooled | 74 |  |
| B | ABP280-D6 | 250 | 73 |  |
|  | ABP279-B5 | 100 | 72 |  |
|  | ABP280-A9 | 250 | 69 |  |
| A | ABP275-F12 | Pooled | 68 |  |
| B | ABP279-F9 | 100 | 68 |  |
| A | ABP272-E11 | Pooled | 67 |  |
|  | ABP277-H1 | Pooled | 65 |  |
|  | ABP271-C6 | Pooled | 64 |  |
|  | ABP274-H3 | Pooled | 64 |  |
|  | ABP271-G4 | Pooled | 63 |  |
| C | ABP282 PL1-G4 | 100 | 59 |  |
| B | ABP279-G6 | 100 | 59 |  |
| A | ABP276-E4 | Pooled | 58 |  |
|  | ABP272-D7 | Pooled | 55 |  |
|  | ABP276-A1 | Pooled | 52 |  |
| N/A | CBS7435 | N/A | 28 |  |
| A | ABP274-D8 | Pooled | 27 |  |
| N/A | ABP234 | N/A | 27 |  |


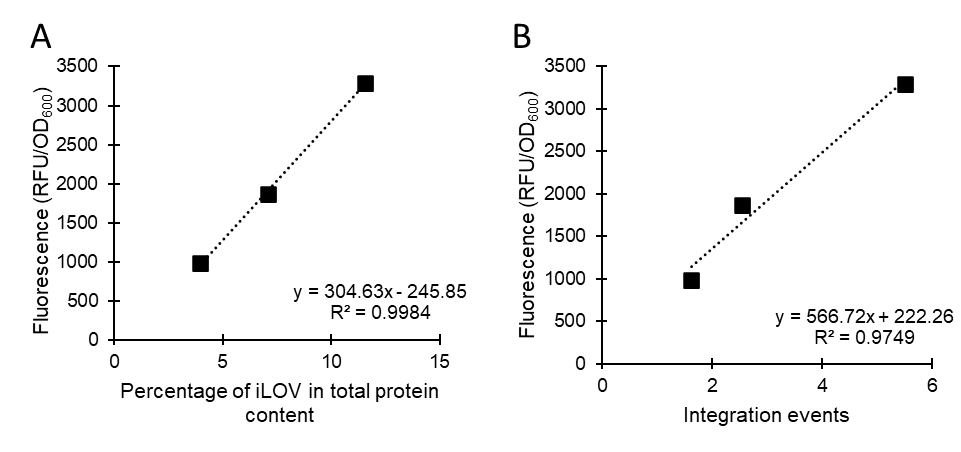


Fig. S1. Relationship between iLOV fluorescence, expression and integration events in *P. pastoris.*

**A.** iLOV expression as a percentage of total protein expression plotted against fluorescence normalised by OD_600_. Briefly, Coomassie stained SDS-PAGE gels were analysed for percentage expression of gene of interest of total cellular protein using the Fiji ImageJ software (Schindelin *et al.*, 2012). The full lane of each sample was plotted as a histogram, and the area under the curve for each peak estimated. The peak corresponding to the gene of interest was divided by the sum of all peaks within the lane for a relative comparison. **B.** Integration events (copy number) of iLOV plotted against fluorescence normalised to OD_600_.


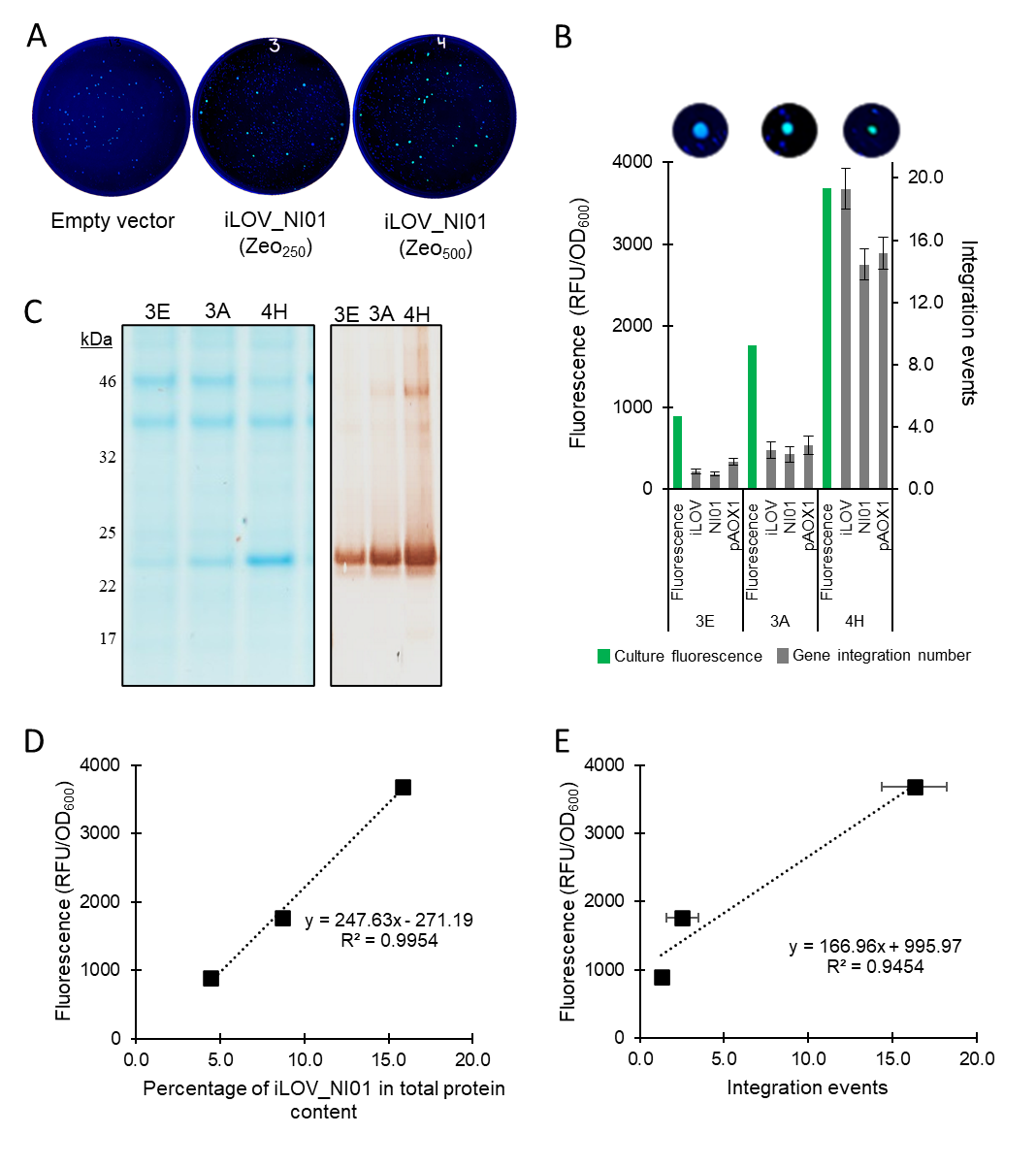


Fig. S2. MTP method for identifying a *P. pastoris* strain expressing iLOV_NI01.

**A**: Induced colonies compared to empty vector control. **B**: Comparison of picked colony with culture fluorescence and gene integration. **C**: Western blot (anti-iLOV) of iLOV_NI01 expression levels in strains analysed in B. **D**: **.** iLOV_NI01 expression as a percentage of total protein expression plotted against fluorescence normalised by OD_600._ Analysed as described in the legend to Figure S1. **E**: Integration events (copy number) of iLOV_NI01 plotted against fluorescence normalised to OD_600_.

Fig. S3. Gating strategy used for identification of iLOV postive cells.

The iLOV population was defined by the exclusion of debris based on FSC-A/SSC-A “Cells” gate, then exclusion of doublet cells using the FSC-A/FSC-W “Single Cells” gate and then the iLOV B530 30-A/ SSC-A “iLOV_N01+” gate was set according to the +ve control sample (iLOV-expressing cells, Fig. 1).


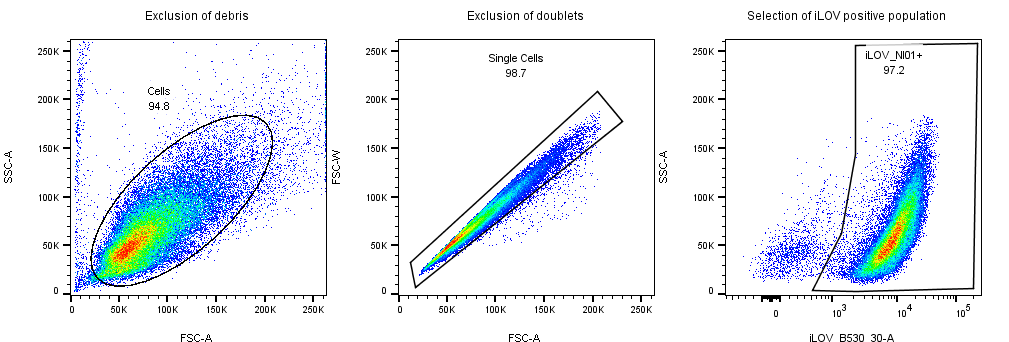


Fig. S4. Representative gating strategy for single cell selection of clones with improved expression of iLOV.

The iLOV population was defined by the exclusion of debris based on FSC-A/SSC-A “Cells” gate, then exclusion of doublet cells using the FSC-A/FSC-H “Single Cells” gate and then the iLOV B530 30-A/ SSC-A “iLOV_N01+" gate was set according to ABP269 to select cells with the highest fluorescence intensity (i.e. 0.15% of the library), resulting in improved iLOV_NI01 expression relative to the benchmark strain ABP269.


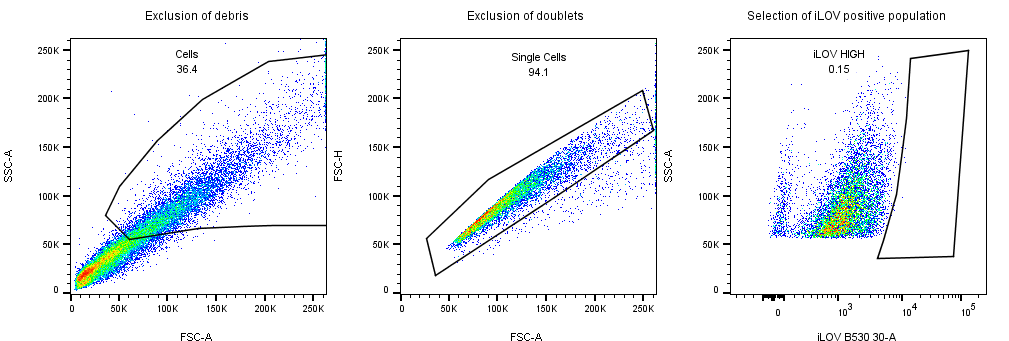


Fig. S5. Full results from liquid screen of FACS hits.

*In vivo* fluorescence (RFU / OD_600_) of all FACS hits normalised to the original ABP269 control strain (green). A total of 90 cell lines selected by FACS were further screened. The five most fluorescent cell lines for each codon optimisation (i.e., 45 clones) were selected from Library A while from Library B it was the five most fluorescent cell lines from each Zeocin concentration (i.e., 15 clones). From Library C, the twenty-five most fluorescent cell lines from Library C were selected. As controls, five cell lines sorted from the benchmark strain ABP269 population were also selected. Wildtype CBS7435 and BSYBG11 were grown alongside to show iLOV-expression resulted in fluorescence above cellular autofluorescence.


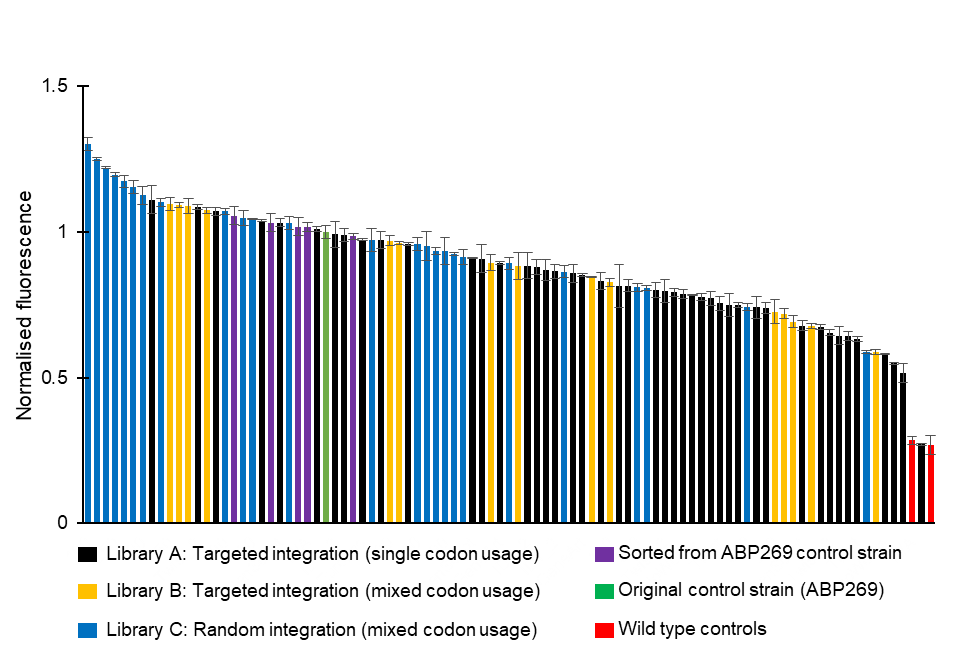


Fig. S6. SDS-PAGE analysis of IMAC purifications of His-tagged iLOV and iLOV_AMP fusions.

4-12 % Bis-Tris Coomassie-stained SDS-gels. iLOV_AMP fusions were purified from cell lysates using 5 mL HisTrap FF Crude columns. Elution was performed by washing with 5 column volumes of 400 mM Imidazole. AMP ID shown at top of gel. Bands of expected size of the iLOV_AMP fusion indicated by arrow.


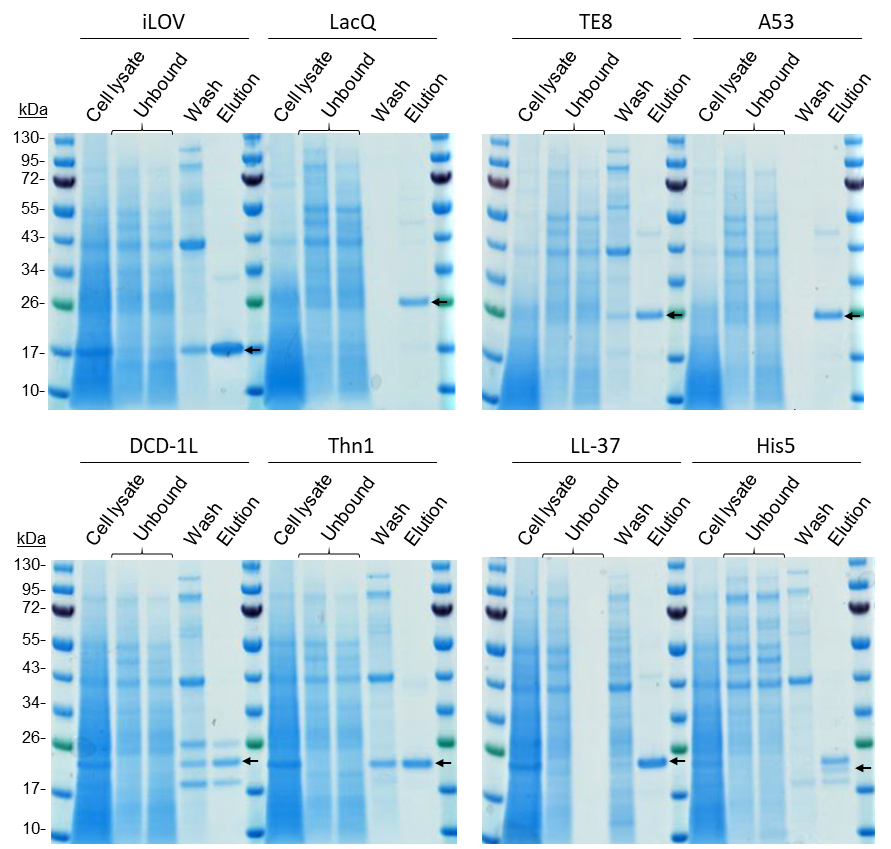


Fig. S7. SDS-PAGE analysis of rEK activity against iLOV and iLOV_LL37.

4-12 % BisTris Coomassie-staind SDS-gel showing purified iLOV and iLOV in the presence of rEK. No cleavage of iLOV is observed, whilst cleavage of purified iLOV_LL37 is observed after incubation with rEK at room temperature over 16 hours to obtain iLOV_residual rEK cleavage site and free LL37.


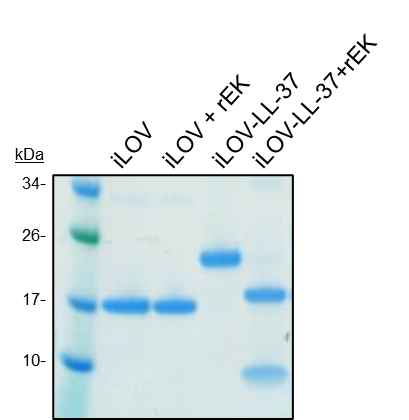

Supplement: Supplementary file 1 — Table S1. Relationship between iLOV_NI01 fluorescence normalised by OD600 and NI01 protein yield in strain ABP290 relative to ABP269. Table S2. Sequences of ABPs. Table S3. Primers used for qPCR. Table S4. Cytometer configuration. Table S5. List of the iLOV‐linker‐Enterokinase‐Site‐AMP amino acidic sequences (AMPs sequences highlighted in grey) Table S6. Strains performance summary. Production yield of NI01 was determined for production strains ABP290 and the ABP269 control (in bold). Fig. S1. Relationship between iLOV fluorescence, expression and integration events in P. pastoris. Fig. S2. MTP method for identifying a P. pastoris strain expressing iLOV_NI01. Fig. S3. Gating strategy used for identification of iLOV postive cells. Fig. S4. Representative gating strategy for single cell selection of clones with improved expression of iLOV. Fig. S5. Full results from liquid screen of FACS hits. Fig. S6. SDS‐PAGE analysis of IMAC purifications of His‐tagged iLOV and iLOV_AMP fusions. Fig. S7. SDS‐PAGE analysis of rEK activity against iLOV and iLOV_LL37. [file MBT2-15-2126-s001.docx]
